# Reward Dependence-Moderated Noradrenergic and Hormonal Responses During Noncompetitive and Competitive Physical Activities

**DOI:** 10.3389/fnbeh.2022.763220

**Published:** 2022-04-26

**Authors:** Zsófia Nagy, István Karsai, Tamás Nagy, Emese Kátai, Attila Miseta, Gábor Fazekas, András Láng, Ferenc Kocsor, János Kállai

**Affiliations:** ^1^Department of Laboratory Medicine, Medical School, University of Pécs, Pécs, Hungary; ^2^Sport and Medicine Research Group, Regenerative Science, Szentágothai Research Centre, University of Pécs, Pécs, Hungary; ^3^Sports and Physical Education Center, Medical School, University of Pécs, Pécs, Hungary; ^4^Department of Vascular Surgery, University of Pécs, Pécs, Hungary; ^5^Institute of Psychology, University of Pécs, Pécs, Hungary; ^6^Department of Behavioral Sciences, University of Pécs, Pécs, Hungary

**Keywords:** reward dependence, temperament and character inventory - revised (TCI-R), neuroendocrinological response, beep running test, competitive condition

## Abstract

The aim of this study was to reveal whether increased reward dependence (RD) plays a role in the catecholamine neurotransmitter release and testosterone hormone regulation during physical activities among healthy trained participants. Twenty-two male participants (mean age: 40.27 ± 5.4 years) participated in this study. Two conditions were constructed, namely, a noncompetitive and a competitive running task (RT), which were separated by a 2-week interval. Urine and blood samples were collected prior to and following the running tasks. Noradrenaline (NA), adrenaline (A), dopamine (D), and their metabolites, vanillylmandelic acid (VMA) and homovanillic acid (HVA), were measured from urine, while testosterone levels were analyzed from blood samples. RD was assessed using the Cloninger’s Personality Inventory (PI). Mental health was evaluated using the WHO Well-Being, Beck Depression, and Perceived Life Stress Questionnaires. According to our findings, levels of NA, A, D, VMA, and testosterone released underwent an increase following physical exertion, independently from the competitive condition of the RT, while HVA levels experienced a decrease. However, we found that testosterone levels showed a significantly lower tendency to elevate in the competitive RT, compared with the noncompetitive condition (*p* = 0.02). In contrast, HVA values were higher in the competitive compared with the noncompetitive condition (*p* = 0.031), both before and after the exercise. Considering the factor RD, in noncompetitive RT, its higher values were associated with elevated NA levels (*p* = 0.007); however, this correlation could not be detected during the competitive condition (*p* = 0.233). Among male runners, the NA and testosterone levels could be predicted to the degree of RD by analyzing competitive and noncompetitive physical exercises.

## Introduction

Recently, several types of research focused upon the psychological, hormonal, and neurotransmitter interactions elicited by physical exercise and sporting competitions. Earlier studies have shown that physical exercise influences neurotransmitter and hormone secretions (Kjaer, 1998; Zouhal et al., 2008; Abderrahmane et al., 2013); however, personality, temperamental traits, and social behavior patterns could also have an impact on these levels (Cohen et al., 2009; Croxson et al., 2009; Salamone and Correa, 2012; Määttänen et al., 2013; Dayan et al., 2014; Varazzani et al., 2015; Casto and Edwards, 2016; Kruk et al., 2020). Cloninger’s biosocial theory regarding personality (Cloninger, 1987; Svrakic et al., 1993) introduced, among others, a fundamental reward dependence (RD) temperament, which is associated with different components of competitive exercises. However, its relation with the activity of the catecholamine and hormonal systems is debated (Cloninger et al., 1993; Cloninger et al., 2019).

The complex molecular genetics and neurobiological basis of temperament are relatively well understood and are known to control the activity of catecholamine and monoaminergic neurotransmitter systems. The RD temperament among healthy individuals is closely related to sensitivity for social reward and is associated with the activation of certain neuroendocrine and endocrine systems, such as oxytocine, norepinephrine, and testosterone (Bell et al., 2006; Garvey et al., 1996; Määttänen et al., 2013).

Today, mental and physical health-related studies are focused on the bio-psychological motivation roots of physical and mental fitness and interactions between the personal motivations and the level of the circulating reward-related catecholamines (CATs) and hormones. Both physical and computer games, sports exercises, or social competitive activities associated with temperamental traits facilitate the assessment of these interactions within *in vivo* situations (Hansenne et al., 2002).

The type of reward in competitive social interaction may be material (food and/or money) or social. The social reward may originate from positive verbal or meta-communication signals, intimacy or public respect, official advancement, etc., which confirm an individual’s position in a family, among peer groups, and the capable handling in receiving support during critical problem-solving situations. Individuals with an above than average RD are motivated to be a winner in various situations whose goal is to obtain social or material rewards (Cloninger, 1987; Han et al., 2006). However, the behavioral phenotype regarding RD is determined by the interaction between neurotransmitters, hormones, and current environmental requirements (Cloninger, 1987; Rózsa et al., 2005).

Individuals with elevated RD seek tenable social territory and are prepared to cooperate with the confirming group’s members, emotionally open toward others, agreeable; however, they are defensive in the face of the concurrent competitors who are rivaling for the same rewards (Cloninger et al., 1993; De Fruyt et al., 2000). Cloninger and De Furyt suggested a behavioral phenotype that is part of the secure territory defending behavior (fight and flight syndrome) and is dominated by noradrenergic transmission. The link between RD and noradrenaline has been confirmed in numerous studies which found an association between low basal noradrenergic levels with high RD temperament (Curtin et al., 1997; Ham et al., 2005). However, noradrenaline activity is sensitive to social and physical stress, and the elevation rate of the circulating noradrenaline (NA) plays a role in the psychophysiological preparation of coping with physical and mental stress.

The most investigated psycho-neuroendocrinological agents during mental or physical stress are CATs (dopamine, adrenaline, noradrenaline) and testosterone. Catecholamines influence the secretion of testosterone and other hormones (Kumar et al., 2013; Kolb and Whishaw, 2020). The concentration of circulating CATs increases during exercise in both men and women (Henderson et al., 2007; Zouhal et al., 2008; Balas et al., 2021). Various studies have pointed out the regulatory role of CATs during the effort of physical achievement (Zouhal et al., 2008). For example, the multistage 20-m shuttle run test or other standardized exercises are effective methods to measure aerobic fitness (Leger and Lambert, 1982; Metsios et al., 2008; Paradisis et al., 2014) and trigger a well-defined catecholamine secretion. The concentration of CATs is a strong indicator regarding the individual’s stress sensitivity in trained and untrained individuals and animals as well (Ratge et al., 1986; Eto et al., 2014; Baker and Buchan, 2017).

Studies on temperamental traits showed how to reward dependency and how seeking social gratification play an essential role in the success of aerobic, endurance, and combat power sports performances (Han et al., 2006). Furthermore, the goal of social approval-oriented athletes is to gain social respect from others (Maehr and Nicholls, 1980; Feltz and Ewing, 1987). Exercise can be beneficial regarding mental health; it decreases stress hormones (Budde et al., 2015), promotes a positive mood, improves confidence, encourages social interaction, and supports weight control (Reblin and Uchino, 2008; Swift et al., 2014). Additionally, social approval-oriented athletic behavior provides a buffer against stress (Childs and de Wit, 2014).

Data from the animal and human studies demonstrated enhanced RD following testosterone administration during ongoing competitive tasks (van Honk et al., 2004). Testosterone has a diurnal variation; the release is highest in the morning and decreases over the day until the afternoon. Its level is influenced by acute or prolonged high-intensity exercise conditions. Acute intensive activities elevate levels of testosterone (Sutton et al., 1973; Cumming et al., 1986), while prolonged exercises decrease (Tanaka et al., 1986) the level of testosterone. In accounting for diurnal rhythm, a testosterone index can be used for an individual temperamental trait to assess RD (Määttänen et al., 2013); social, affiliative behavior; and the involved status competition (Daitzman et al., 1978; Dabbs, 1990; Eisenegger et al., 2011).

In the course of competitive activities, testosterone elevation promotes the successful outcome of the task challenge (Carre et al., 2013) and enhances motivation to win a social and/or monetary reward (van Honk et al., 2004; Coates and Herbert, 2008; Mehta et al., 2015; Welker et al., 2015). The testosterone hormone is linked to the regulation of catecholamines and advances certain behaviors, such as reward-seeking, status-seeking, and competitive activity (Archer, 2006). Furthermore, testosterone influences the competitive sports activity among humans; however, the effect is indeed selective and dependent upon the social and physical challenges, personal motivation factors of the individuals, and, specifically, the characteristics of a given sports activity (Casto and Edwards, 2016).

Previous research has ushered in supportive evidence regarding forms of both noradrenaline and adrenaline, influencing the emotional element of social behavior (Åstrand and Rodahl, 1986). However, other CATs, including dopamine, also participate in the regulation of social behavior and RD (Kim et al., 2006; Skuse and Gallagher, 2009; Lombardo et al., 2012). Thus, the NA action for RD cannot effectively be viewed in isolation since it is linked to dopamine functions.

The primary role of this study was to reveal associations between the psychometrically defined RD and the physical and mental stress-related testosterone and CATs responses in a group of physically trained healthy athletes. Physical and mental stress was triggered by a well-defined standard task, the intermittent running exercise, performed in both noncompetitive and competitive conditions. Blood and urinary samples for CATs, CAT’s metabolites, and testosterone were obtained immediately before and after the training.

Based on the suggestions of Zouhal et al. (2008) and Kruk et al. (2020), we examined a group of middle-aged, healthy, nonprofessionally trained male athletes actively participating in both noncompetitive and competitive running conditions. We hypothesized that the RD temperament as a biological-based heritable response pattern to physical and social stress is associated with the CATs and their metabolite levels in urine.

Additionally, we believe that the induced psychoneuroendocrinological responses are transmitted by the modulation effect of RD both in noncompetitive and competitive activities, albeit, in various CATs patterns. We hypothesized that the competition triggers CATs or testosterone response regarding participants who are sensitive to the social and peer reward. This psychoneuroendocrinological effect is manifested in higher concentrations of testosterone. However, during noncompetitive activities, lower-level CATs response patterns may be expected. Considering the noradrenaline and testosterone interactions, other CATs, adrenaline, and dopamine levels will be monitored and explored shortly.

## Materials and Methods

### Subjects and Experimental Design

All subjects were informed regarding the procedures including the potential risks of the experiments prior to obtaining written informed consent. All procedures were approved by the Regional Committee for Research Ethics of the Locale State University (ref. No.: 7162/2018). Twenty-two male participants (mean age: 40.27 years; SD: 5.4; range: 31–49 years) were recruited for the study, who were selected by the following criteria: a history of regular training (running, cycling, and swimming) at least three times a week for a minimum of 45 min/occasion, participation in competitions at least once a year, no known acute or chronic disease present, no regular use of prescription medication, and no obesity. All participants were examined by a Doctor of Medicine, and ECG, including monitoring blood pressure, heart rate, and a general health questionnaire regarding sports medicine were all performed and duly noted. Subjects were asked to respond to questionnaires (included the Temperament and Character Inventory – R version) online before the time of the physical activities.

The experimental setup was designed to feature two phases: In the first phase, volunteers were assessed individually by a running beep test in a noncompetitive activity, while in the second phase, subjects participated in a running beep test (RT) competition in which they were split into 2–4 individuals per team. In the second phase, other participants were invited and encouraged to support their fellow athletes. The phases were separated into 2-week periods. The main reason for the order of the tests was practical feasibility. We had to assess the physical performance/capacity in the noncompetitive condition in order to form competitive groups. Once we had the data, individuals with comparable physical performance levels were enrolled in subgroups to create a competitive situation (in which every participant had a reasonable chance to win his/her group).

During the physical phases, volunteers were asked to avoid hormone-containing menu items (e.g., soya, legumes, and milk), not to consume alcohol or use recreational drugs before the day of the experiment, and to fast for a minimum of 2 h as required before the survey.

Only water consumption was allowed. All participants were asked to restrain from exercising during the day before their assessment. All RTs were executed during the evening hours. Blood and urine samples were collected immediately before and following the tests. Questionnaires were also completed within 15 min before and immediately following the RT.

### Questionnaires

Before the RT, a personality test questionnaire pocket was administered. The RD is an essential factor regarding temperament traits and is measured using the Temperament and Character Inventory – R version (TCI-R, with 240 items, response range 0–4), which contains novelty seeking, harm avoidance, RD, and persistence factors (Cloninger et al., 1999; Rózsa et al., 2005).

Considering the aim of this study during our investigation, only the RD factor served as the object of the next analysis. Furthermore, the current health status of participants screened by the Perceived Stress Questionnaire, with fourteen items (response range 0–4; Cohen et al., 1983; Stauder and Konkolÿ-Thege, 2006), WHO Well-Being Questionnaire short version with five items (response range 1–5; Susánszky et al., 2006; Topp et al., 2015), and Beck’s Depression Scale short version with ten items (response range 1–4; Beck et al., 1961; Rózsa et al., 2001). All participants’ health-related questionnaire data were conferred with the national-based mean and standard deviation scores, and each subject was to be found within the healthy range of the normative sample.

### Blood Samples

Before and immediately following, the RT venous blood collections were performed in suitable vacutainers; tubes containing potassium ethylenediaminetetraacetic acid (K-EDTA) were used for testing cellular blood parameters. Tubes containing sodium-fluoride (NaF) were used for plasma glucose and lactate analysis, while native tubes were used to obtain serum in support of the routine laboratory blood tests.

All samples were transferred to the laboratory within 2 h, in which both plasma and serum were separated using centrifugation (15 min, room temperature, 1,500 *g*). Blood cell parameters were quantified in a multi-parameter automatic hematology analyzer, Sysmex XN-Series 9000 (Sysmex Corporation, Kobe, Japan). Plasma and serum parameters were measured using the Cobas 8000 Modular Analyzer (Roche Diagnostics, GmbH, Mannheim, Germany), while testosterone levels were measured using the ARCHITECT i2000SR Analyzer (Abbott Diagnostics, Abbott Park, IL, United States) in strict accordance with the manufacturer’s recommended guidelines.

### Urine Samples

Middle stream urine was collected before and immediately following RT and stored in native bottles. All samples were checked by a rapid test (Cybow 10). At the laboratory, urine samples were aliquoted and frozen at −80°C until further use. Following the thaw, dopamine (D), adrenaline (A), and noradrenaline (NA) levels were detected using the Shimadzu Prominence High-Performance Liquid Chromatography (HPLC) system with Antec Decade SDCTM electrochemical detector including the Chromsystems^®^ kit [Chromsystems^®^ from ABL&E-JASCO Hungary, Budapest, Catecholamines in urine – HPLC kit (ref. No.: 6000)] and reverse phase column (Chromsystems^®^ from ABL&E-JASCO Hungary, Budapest, ref. No.: 5100) in strict accordance with the manufacturer’s recommended guidelines. The flow rate was 1.3 ml/min at room temperature. Vanillylmandelic acid (VMA) and homovanillic acid (HVA) levels were detected using the same system such as the Chromsystems^®^ kit [Chromsystems^®^ from ABL&E-JASCO Hungary, Budapest, VMA, HVA, 5-OHIAA in urine – HPLC kit (ref. No.: 1000/B)] and reverse phase column (ref. No.: 1100/B) in strict accordance with the manufacturer’s recommended guidelines. The flow rate was 0.8 ml/min at room temperature. All data were evaluated using the LabSolution program.

### Procedure

The laboratory indicators were measured using a standard clinical method regarding the participants’ blood and urinary samples, before and immediately following the noncompetitive and competitive RT. According to the health state-related anamnesis, the laboratory evidence including the physical examination substantiated and validated, all participants were indeed declared healthy. Recorded biomarkers can be found in Supplementary Figure 1, which supported the healthy stage and/or the executed RT.

In consideration of physical exertion, the multistage 20-m shuttle run test was used. The noncompetitive activity involved running a distance of 20 m, synchronized to audio sounds, which progressively increase in tempo each minute, in which time was gradually reduced. The RT lasted until total exhaustion (Leger and Lambert, 1982; Leger and Gadoury, 1989). The competitive task contains the same exercise; however, the RT was relocated to a competitive environment in which the running was performed in front of a rewarding social context. Heart rate was registered as formerly measured, during exercise, and following the activity, using the Polar Team Pro System. Volunteers were asked to don transmitters to the middle of their chest under the tip of the xiphoid process. Blood and urinary sample were immediately taken prior to and following completing noncompetitive and competitive activities.

### Data Analyses

Statistical analyses were run using IBM SPSS Statistics (version 22.0). Means and standard deviations were calculated. To test the main effects of sampling time (pre- vs. post-physical exercise) and competition condition (noncompetitive vs. competitive condition), we used repeated measures ANOVAs. Both sampling time and competition are within-subject effects. No within-subject effects were tested in the repeated measures ANOVAs. Because we had only two levels in the analyses and no between-subjects effects were tested, homogeneity of variances and the assumption of sphericity are both met by default. Evidence (Stiger et al., 1998; Schmider et al., 2010) shows that ANOVAs are robust to violations of normality, and therefore, normality was not tested. The required sample size for repeated measures ANOVAs was calculated using G*Power 3.1 (Faul et al., 2009). Setting the threshold for rejecting the null hypothesis to α = 0.05, a sample size of at least 27 participants was calculated to detect medium-size effects (partial η2 = 0.06) with an adequate power of 0.80. For detecting small effects (partial η2 = 0.01), a sample size of at least 164 participants would have been required to reach a power of 0.80. Therefore, our study can be considered as underpowered in detecting small and medium effects.

Data analyses were divided into two sections. The first section controlling the validity of the measuring paradigms focused on the analyses of the effect regarding the condition and the pre- and post-measured scores on selected neurotransmitters, metabolites, and testosterone. The second part focused on the role of the RD temperament effects on detected transmitter reactivity in competitive or noncompetitive activities. The dynamical changes of the circulated neurotransmitters, their metabolites, and testosterone levels were analyzed using a stepwise regression analysis separately in noncompetitive and competitive conditions. The dependent variables were defined as the total score of the RD factor, and the pre- and post-ratios of neurotransmitters in noncompetitive and competitive conditions were included as predictors. Indeed, 22 men volunteered for the study, but only 18 individuals finished the tests completely. Four persons had to be excluded either because they missed some of the RT sessions or the blood or urine samples could not be collected.

## Results

Participants’ health-related variables are in Table 1. The runners were asked to perform noncompetitive and competitive running activities in which the same neurotransmitters were analyzed in the pre-phase and post-phase regarding the RT. In all cases, in the noncompetitive condition, the within-subject analysis showed elevation between pre-phase and post-phase of the exercise-triggered neurotransmitter concentrations (the effect sizes are medium or large), except for HVA. In the case of the HVA, the concentration to the end of the RT is decreased. In the competitive condition in all cases, the level of the CATs concentration is elevated except the HVA where the concentration of the D metabolite is decreased. However, in this competitive condition, the change of the testosterone and VMA concentrations is not significant (Supplementary Figure 3).

**TABLE 1:** Participant’s health-related variables.

	*n*	mean (SD)
**Participants’ health-related variables**		
***Health-related scores and temperamental trait***		
Age	22	40.27 (5.4)
Perceived stress	22	21.0 (5.2)
WHO well-being	22	11.5 (3.0)
Beck depression	22	11.3 (2.4)
TCI reward dependence	22	96.5 (15.2)

***Physical and physiological performance scores following non-competitive and competitive activities***		

Non-competitive RT (in meter)	21	2514.8 (569.9)
Competitive RT (in meter)	18	2915.7 (524.4)
Non-competitive RT max pulse (bit/min)	21	182.1 (14.8)
Competitive RT max pulse (bit/min)	18	187.0 (14.5)
Non-competitive relative VO_2_ maximum (mL/kg/min)	21	43.5 (5.4)
Competitive relative VO_2_ maximum (mL/kg/min)	18	46.2 (4.8)
Non-competitive plasma lactate (in mmol/L) Competitive plasma lactate (in mmol/L) Non-competitive glucose (in mmol/L) Competitive glucose (in mmol/L)	21 18 21 18	11.4 (2.5) 10.9 (3.6) 7.9 (1.1) 7.7 (1.3)

*Descriptive characteristics represent the sample for health-related indexes, reward dependence score, physical performance, and physiological data.*

Results of repeated measures ANOVAs are presented in Table 2. In addition to the significant main effect regarding the pre-RT and post-RT levels of NA, a significant interaction with large effect size (partial η^2^ = 0.318) was found between the pre-phase and post-phase biomarker levels and the condition of the task. This implies that the level of NA increased from pre-task to post-task; however, the increase was significantly higher during the competitive condition. Concerning A and D, the RT had a significant effect only upon their levels; both increased from pre-task to post-task.

**TABLE 2:** The effect of conditioning, RT (biomarker level from pre-RT to post-RT), and their interaction (RT × condition) on neurotransmitters.

CATs hormone	Condition (non-comp and comp)			RT (levels from pre to post task)			RT × Condition		
	*F*	*p*	Partial η^2^	*F*	*p*	Partial η^2^	*F*	*p*	Partial η^2^
NA	1.211	0.286	0.067	**33.615**	**<0.001**	**0.664**	**7.934**	**0.012**	**0.318**
A	0.638	0.436	0.036	**14.944**	**0.001**	**0.468**	2.924	0.757	0.006
D	4.198	0.056	0.198	**21.947**	**<0.001**	**0.564**	2.155	0.160	0.113
Testo	1.069	0.316	0.059	**20.618**	**<0.001**	**0.548**	**6.554**	**0.020**	**0.278**
VMA	**4.769**	**0.043**	**0.219**	**7.272**	**0.015**	**0.300**	0.001	0.973	0.000
HVA	**5.535**	**0.031**	**0.246**	**19.149**	**<0.001**	**0.530**	1.698	0.210	0.091

*Results of repeated measures ANOVAs (n = 18).*

*Significant effects are highlighted in bold.*

**FIGURE 1:**
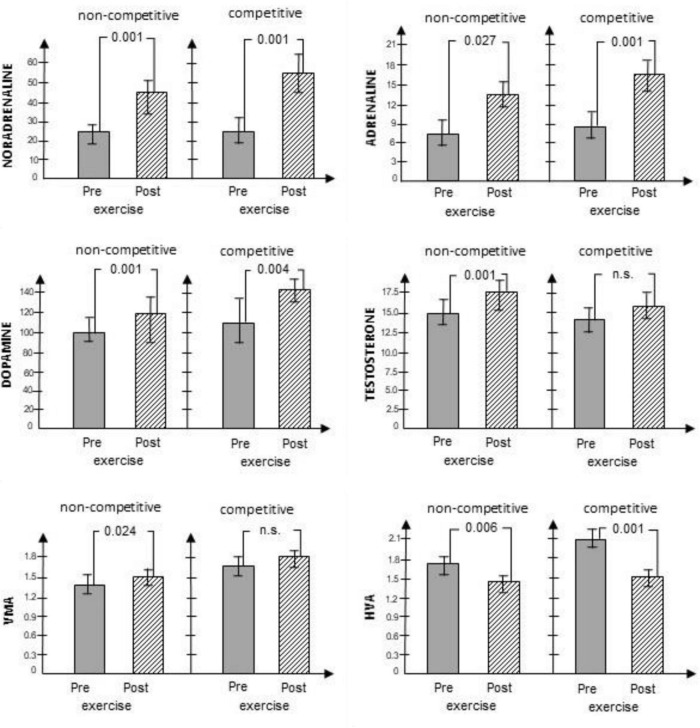
Changes in CATs, their metabolites and testosterone levels measured prior to and immediately following the RT in noncompetitive and competitive conditions. SD levels are marked, *p* levels are numbered above the bar diagrams. NA in nmol/L, A in nmol/L, D in nmol/L, testosterone in nmol/L, VMA in μmol/L, HVA in μmol/L.

In reviewing the levels of testosterone, beyond the significant main effect of the RT, a significant interaction with large effect size (Partial η^2^ = 0.278) was found between pre-task and post-task value and condition. This infers that the level of testosterone increased from pre-task to post-task; however, the increase was significantly higher in the noncompetitive condition. In consideration of VMA and HVA, both the main effect of the RT and the main effect of the conditions were significant. However, no significant interaction was found. In the case of VMA, this suggests that its levels were higher in the competitive condition when compared with the noncompetitive condition, and its levels showed a significant increase from pre-task to post-task. Levels of HVA were also higher in the competitive condition, as compared with the noncompetitive condition; yet, its levels showed a significant decrease from pre-task to post-task. Results are depicted in Figure 1.

In the second step of the data analysis, the association of the RD, the examined biomarkers were accomplished. Considering the present data depicted in Table 3, the degree of the RD in men can be predicted by the catecholamine and testosterone concentration while an individual is completing various physical activities, yet differ in some respects, e.g., its circumstances.

**TABLE 3:** NA and testosterone concentrations may likely be predictors for RD.

RD and CATs and testosterone	*R*	*F*	β	*t*	sig.
NA noncompetitive post/pre ratio	0.586	9.39	0.586	3.06	**0.007**
Testosteron competitive post/pre ratio	0.519	5.53	−0.519	−2.35	**0.033**

*The analyzed data originate from the former scheduled testosterone levels from blood and neurotransmitters and their metabolites from urine samples before and following noncompetitive and competitive RT. The D, A, NA, testosterone, VMA, HVA, and noncompetitive and competitive pre-ratio and post-ratio were the objects of the analysis. Significant values are highlighted in bold. The nonsignificant associations are not presented here.*

**FIGURE 2:**
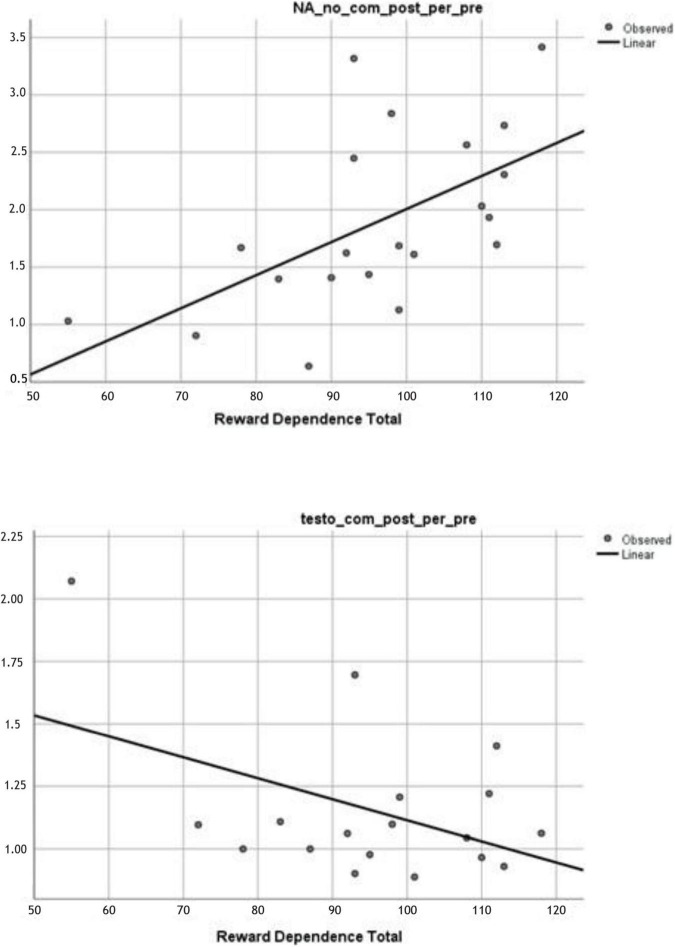
Up: NA post/pre exercise ratio (y-axis) results in noncompetitive condition as a function of RD scores (x-axis), *p* = 0.007. Down: Testosterone post/pre ratio (y-axis) results in competitive condition. It is negatively associated with the RD scores (x-axis), *p* = 0.033.

In noncompetitive RT, the elevation of the pre-ratio and post-ratio of NA is associated with a high score in RD. In contrast, a similar association was not detected in competitive conditions. In competitive conditions, a testosterone response and RD associations were found which indicated that the testosterone pre-ratio and post-ratio are lower in an individual with a high rate of RD. In summary, among individuals with a high degree of RD and actively engaged in a competitive situation, the testosterone level in the final phase of the RT when compared with the starting phase, the level of free concentration shows a significantly attenuating tendency. Results are illustrated in Figure 2, and the nonsignificant associations are presented in Table 4.

**TABLE 4:** Nonsignificant associations of measured biomarkers with RD scores.

	*R* square	Sig.
NA_com_post/pre	0.088	0.233
Testo_no_com_post/pre	0.012	0.65
A_no_com_post/pre	0.084	0.203
A_com_post/pre	0.009	0.702
D_no_com_post/pre	0.098	0.167
D_com_post/pre	0.08	0.27
VMA_no_comp_post/pre	0.044	0.36
VMA_comp_post/pre	0.006	0.751
HVA_no_com_post/pre	0.001	0.874
HVA_comp_post/pre	0.015	0.633
*n = 18*		

*Noncompetitive and competitive pre-ratio and post-ratio were the objects of the analysis. Dot plot diagrams are found in Supplementary Figure 2.*

## Discussion

Our study was based on the main hypothesis in which an individual with high RD during different exercise-triggered situations responds with different CATs and hormone (testosterone) patterns. Our results indicated the elevation levels of the analyzed CATs and their metabolites (except HVA), and testosterone is relatively independent of the applied noncompetitive or competitive condition. Notably, these different response tendencies to physical stress have been confirmed by previous studies as well (Sutton et al., 1973; Wheeler et al., 1994; Määttänen et al., 2013; Casto and Edwards, 2016; McMorris, 2016; Kruk et al., 2020). However, we found that a tendency regarding testosterone is lower in the case of the competitive condition. VMA levels in the within-subject analysis showed a post-task enhancement. HVA levels following RT in both noncompetitive and competitive conditions when compared with the pre-task levels were decreased. A previous study showed that the concentration of HVA is strongly associated with dopamine levels in the brain and, in a majority of the cases, specifically in the brain stem and hypothalamus (Hasegawa et al., 2000); however, caution is recommended in this regard (Kopin et al., 1988). Therefore, this HVA level reduction deserves thoughtfulness in the interpretation of the obtained results. HVA elevated levels are frequently detected during increased stress responsiveness, as depicted in anxiety, depression, and schizophrenic cases (Breier et al., 1993; Marcelis et al., 2006; Kurita et al., 2015). In our study, HVA showed that during the noncompetitive physical activity, the stress reactivity at the end of the RT is decreased. Based on our present data, it can be stated that the RTs with the applied parameters can affect the D/NA stress reaction system among psychopathology-free healthy individuals. NA is one of the main messengers that regulates arousal, attention, emotional state, learning, memory, and stress response (Aston-Jones and Cohen, 2005). Considering the role of NA in the neurocognitive mechanism of the salience expectancy processing, previous data (Li et al., 2017) suggest that RD can be considered as a sensitivity index to the reward-based salience when an individual approaches a goal or completes a task in which the significance of the reward may be augmented. The reward processing can be accounted as a state-dependent complex neural system that engages with the trial, trial learning in skills training and shaping of social behavior, and is controlled by the physiological state and many situational factors (Schonberg et al., 2007; Cohen et al., 2009; Dayan et al., 2014). The reward is a continuum and is processed in several phases in which the upcoming event is evaluated by cost and effort balance. The undertaking for action and the execution of the goal-directed behavior involve a dual mechanism. The first is a decisive phase in which the individual evaluates the rate of the expected reward. This phase is dominated by dopaminergic reward evaluated functions. The second phase focuses on the execution of the action, controlled by the noradrenergic system in which the upcoming effort decreases the value of the expected rewards. This reward/effort trade of model (Croxson et al., 2009; Salamone and Correa, 2012; Varazzani et al., 2015) may be adapted to understand the biological and motivational bases of sports activities. The noradrenergic transmission is a dominant agent in the regulation of social behavior frequently linked to RD. However, the tendency and intensity of the association of the psychometrically defined RD and NA concentration are controversial. In an earlier study, urinary MHPG (3-methoxy-4-hydroxyphenylglycol, a NA metabolite concentration) was measured, and a strong positive relationship between RD and a basic level of MHPG (Curtin et al., 1997) was found. In another study, healthy volunteers were assessed using Cloningers’ PD scales and MHPG, and over 24 h, urine was collected and assessed. This study reported a reverse association between RD and MHPG concentration (Garvey et al., 1996). In our experiments, the volunteers consisted of middle-aged, moderately physically trained men. The analysis of urine and serum samples indicated the current state-dependent amount of CATs and testosterone. Our data support the hypothesis in a relationship that exists between the RD and the NA neurotransmitter and is manifested during intensified forms of activity. However, this association diminished when the effort is larger, and the competition is facing an audience while competing with other participating members. Our data support Cloningers’ suggestion, that is, temperament is a catecholamine system-related behavioral and emotional disposition of how an individual learns, copes, and adapts to various changes in his/her environment. Additionally, we analyzed the association between RD and the pre-/post-ratios regarding the dynamic changes of the CATs and testosterone. In a stepwise regression analysis, two condition-dependent associations were revealed. In the noncompetitive condition, the rate of NA concentration change was found to be associated with RD. Additionally, the pre-test and post-test ratios of NA were indeed predictive regarding the high score of RD. In the competitive condition, we found no association between CAT’s pre- and post-ratios and RD. However, an inverse correlation was found between the dynamical changes of testosterone and RD scores. The within-subject analysis demonstrated that the testosterone concentration is strongly increased following noncompetitive conditions, and a small increase was found in competitive RT conditions. This finding is aligned with earlier results which suggest challenges that increase the level of motivation and elevate the concentration of testosterone, primarily among men (Archer, 2006; Carre et al., 2010; Wood and Stanton, 2012). However, other factors including fitness, motivation, and personality can influence the concentration of testosterone during competitive RT completion (Schultheiss, 2007; Maner et al., 2008; van der Meij et al., 2010). Despite this data, in our findings, the dynamical index (pre-ratio/post-ratio) regarding the testosterone concentration showed an attenuation tendency among those who had high RD scores. Competitive, elite sports activity is a major interest referencing stress-related neuroscientific research. Studies investigate the cost–benefit balance of competitive physical activity and explore advantageous and disadvantageous consequences. According to the current view of the psychophysiological allostasis, the competition-associated anxiety inhibits catecholamine release; the hormonal system interaction increases the perception of negative emotions and disturbs the skill automatism and the attention concentration in rivaling situations (Wheeler et al., 1994; Ong and Chua, 2020). Our present results show how testosterone concentration is increased in noncompetitive and competitive RT conditions following exercise. However, the ratio of change is dependent upon an individual temperament bias, specifically with regard to the RD. The presented data showed that high RD is associated with a low concentration of testosterone following a competitive task. The reason for this association has not yet been clarified. Suitable interpretation may be found within the neuroendocrinology of reward and behavioral dysregulation. Welker et al. (2015) suggested in a hypothesis that stress hormones and the lactate level bear a crucial role in the regulation of the interaction of reward and testosterone. According to these researchers, analysis of the cortisol and lactate levels may be an adequate means to understand the mechanism regarding the high RD and testosterone attenuation during the completion of the activity. High RD may be associated with the hemodynamical changes during physical stress which is affected by the metabolization of cortisol and lactate levels and the rate of the subject cardiovascular effort. In summary, we found evidence of how the central emotional arousal is elevated when a subject performs an activity involving physical stress. Following noncompetitive physical stress, NA levels significantly increased. However, in competitive conditions, the RD temperament is associated with the concentration of testosterone after RT. In this condition, the elevated RD is associated with a low level of serum testosterone when a runner has finished his race. Contrary, sportsmen with lower RD scores have a higher and maintained concentration testosterone after finishing their race.

## Conclusion

We focused on the individual variability regarding dynamic changes between the pre-exercise and post-exercise CATs and testosterone in competitive and noncompetitive conditions. We found that RD scores were associated with NA and testosterone levels which could be indicated in that this temperamental trait affects the noradrenergic system stress response including testosterone release in the case of physical exertion in different psychical circumstances.

### Study Limitations

In this study, only male participants were enrolled. The interpretation of the results concerned with the psychological functions and their associations with testosterone and the main catecholamines; however, the complex biochemical interaction between several catecholamines and other temperament factors has not been evaluated. The interaction between temperament and character factors was not the objective of this study. The role of the found association in the participants’ sociobiological adaptation has not been tested. The limited number of participants did not allow to reveal the interaction and the role of the dopaminergic transmission in RD. Due to the rigorous selection criteria, despite the relatively small number of persons available, the reported conclusions are tenable based on the found partial η^2^ large effect sizes. However, further examinations are recommended to confirm the results.

## Data Availability Statement

The datasets presented in this study can be found in online repositories. The names of the repository/repositories and accession number(s) can be found in the article/Supplementary Material.

## Ethics Statement

The studies involving human participants were reviewed and approved by Regional Committee for Research Ethics of the Locale State University (ref. No.: 7162/2018). The patients/participants provided their written informed consent to participate in this study.

## Author Contributions

ZN was involved in conceptualization, methodology, investigation, resources, project administration, writing the original draft, editing and supervision. IK was involved in conceptualization, methodology, funding acquisition, writing and supervision. TN, AM, and GF were involved in writing and supervision. EK was involved in investigation and supervision. AL and FK were involved in the statistical analysis. JK was involved in the conceptualization, statistical analysis, funding acquisition, writing the original draft, editing and supervision. All authors contributed to the article and approved the submitted version.

## Conflict of Interest

The authors declare that the research was conducted in the absence of any commercial or financial relationships that could be construed as a potential conflict of interest.

## Publisher’s Note

All claims expressed in this article are solely those of the authors and do not necessarily represent those of their affiliated organizations, or those of the publisher, the editors and the reviewers. Any product that may be evaluated in this article, or claim that may be made by its manufacturer, is not guaranteed or endorsed by the publisher.

## Abbreviations

RD: reward dependence
CAT: catecholamine
A: adrenaline
NA: noradrenaline
D: dopamine
VMA: vanillylmandelic acid
HVA: homovanillic acid
RT: running beep test
MHPG: 3-methoxy-4-hydroxyphenylglycol.
