# Supplementary material for: Reward Dependence-Moderated Noradrenergic and Hormonal Responses During Noncompetitive and Competitive Physical Activities
Source: Front Behav Neurosci. 2022 Apr 26;16:763220. doi: 10.3389/fnbeh.2022.763220 (PMC9087724; doi:10.3389/fnbeh.2022.763220)
Supplement: Supplementary file 1 [file Table_1.DOCX]

**SUPPLEMENT**

**A.**

|  | Non-competition | | | |  | Competition | | | |  |
| --- | --- | --- | --- | --- | --- | --- | --- | --- | --- | --- |
|  | Before |  | After |  |  | Before |  | After |  | Units |
| Sodium | 142.3 | ±1.0 | 143 | ±1.6 |  | 141.8 | ±1.7 | 141.7 | ±1.7 | mM |
| Potassium | 4.3 | ±0.3 | 4.0 | ±0.3 |  | 4.4 | ±0.4 | 4.1 | ±0.4 | mM |
| Calcium | 2.5 | ±0.1 | 2.6 | ±0.1 |  | 2.5 | ±0.1 | 2.5 | ±0.1 | mM |
| Magnesium | 0.9 | ±0.1 | 0.9 | ±0.1 |  | 0.9 | ±0.0 | 0.9 | ±0.1 | mM |
| Phosphate | 1.2 | ±0.1 | 1.6 | ±0.2 |  | 1.3 | ±0.1 | 1.6 | ±0.1 | mM |
| Glucose | 5.3 | ±0.8 | **8.0** | ±1.1 |  | 5.0 | ±0.4 | **7.7** | ±1.3 | mM |
| Lactate | 1.3 | ±0.4 | **11.4** | ±2.6 |  | 1.5 | ±0.8 | **11.0** | ±3.6 | mM |
| Creatinin | 86.7 | ±14.0 | **104.0** | ±15.0 |  | 82.7 | ±15.1 | **100.3** | ±15.8 | µM |
| Creatine-kinase | 237.0 | ±121.2 | **271.8** | ±141.5 |  | 246.2 | ±126.8 | **267.8** | ±132.7 | U/L |
| Lactate dehydrogenase | 373.0 | ±60.0 | **402.6** | ±59.7 |  | 380.3 | ±58.0 | **415.2** | ±72.5 | U/L |
| Total protein | 73.2 | ±6.2 | **77.8** | ±4.3 |  | 75.8 | ±4.3 | **78** | ±4.4 | g/L |
| Albumin | 48.3 | ±3.3 | **50.4** | ±2.3 |  | 49.3 | ±2.4 | **50.6** | ±2.1 | g/L |
| White blood cells | 7.3 | ±1.2 | **11.6** | ±2.3 |  | 7.8 | ±1.6 | **11.2** | ±2.6 | G/L |
| Neutrophils | 3.7 | ±0.9 | **4.7** | ±1.2 |  | 4.1 | ±1.0 | **4.9** | ±1.2 | G/L |
| Lymphocytes | 2.7 | ±0.6 | **5.7** | ±1.4 |  | 2.8 | ±0.7 | **5.1** | ±1.5 | G/L |
| Monocytes | 0.6 | ±0.2 | **0.9** | ±0.3 |  | 0.6 | ±0.1 | **0.9** | ±0.2 | G/L |
| Eosinophils | 0.2 | ±0.1 | 0.3 | ±0.2 |  | 0.2 | ±0.1 | 0.2 | ±0.1 | G/L |
| Basophils | 0.1 | ±0.0 | 0.1 | ±0.0 |  | 0.1 | ±0.0 | 0.1 | ±0.1 | G/L |
| RBC | 5 | ±0.4 | **5.2** | ±0.4 |  | 4.9 | ±0.3 | **5** | ±0.3 | T/L |
| Hgb | 148.2 | ±10.1 | **152.4** | ±8.2 |  | 145.9 | ±7.3 | **149.7** | ±7.8 | g/L |
| Hct | 42.9 | ±3.0 | **45** | ±2.9 |  | 41.7 | ±1.7 | **43.6** | ±2.0 | % |
| Platelet | 250.3 | ±34.3 | **290.4** | ±43.9 |  | 253.7 | ±41.6 | **300.6** | ±55.0 | G/L |
| Carbamide | 5.7 | ±1.1 |  |  |  |  |  |  |  | mM/L |
| Cholesterol | 4.8 | ±0.9 |  |  |  |  |  |  |  | mM/L |
| GOT | 30.1 | ±11.4 |  |  |  |  |  |  |  | U/L |
| GPT | 29.1 | ±16.7 |  |  |  |  |  |  |  | U/L |
| TSH | 3.1 | ±1.4 |  |  |  |  |  |  |  | mg/L |
| CRP | 1 | ±1.0 |  |  |  |  |  |  |  | mU/L |
|  |  |  |  |  |  |  |  |  |  |  |
| *Data are mean±SD* |  |  |  |  |  |  |  |  |  |  |
| *In the case of non-competition n=21, and in competitive condition n=18* | | | | |  |  |  |  |  |  |
| *Carbamide, Cholesterol, GOT, GPT, TSH and CRP were measured only at the baseline* | | | | | | |  |  |  |  |

**Supplement/ Figure. 1.** Serum blood parameters before and after exercises. Significant associations are in bold (p<0.05 before RT vs. after RT).
