# Supplementary material for: Reward Dependence-Moderated Noradrenergic and Hormonal Responses During Noncompetitive and Competitive Physical Activities
Source: Front Behav Neurosci. 2022 Apr 26;16:763220. doi: 10.3389/fnbeh.2022.763220 (PMC9087724; doi:10.3389/fnbeh.2022.763220)
Supplement: Supplementary file 2 [file Image_1.pdf]

SUPPLEMENT

B.

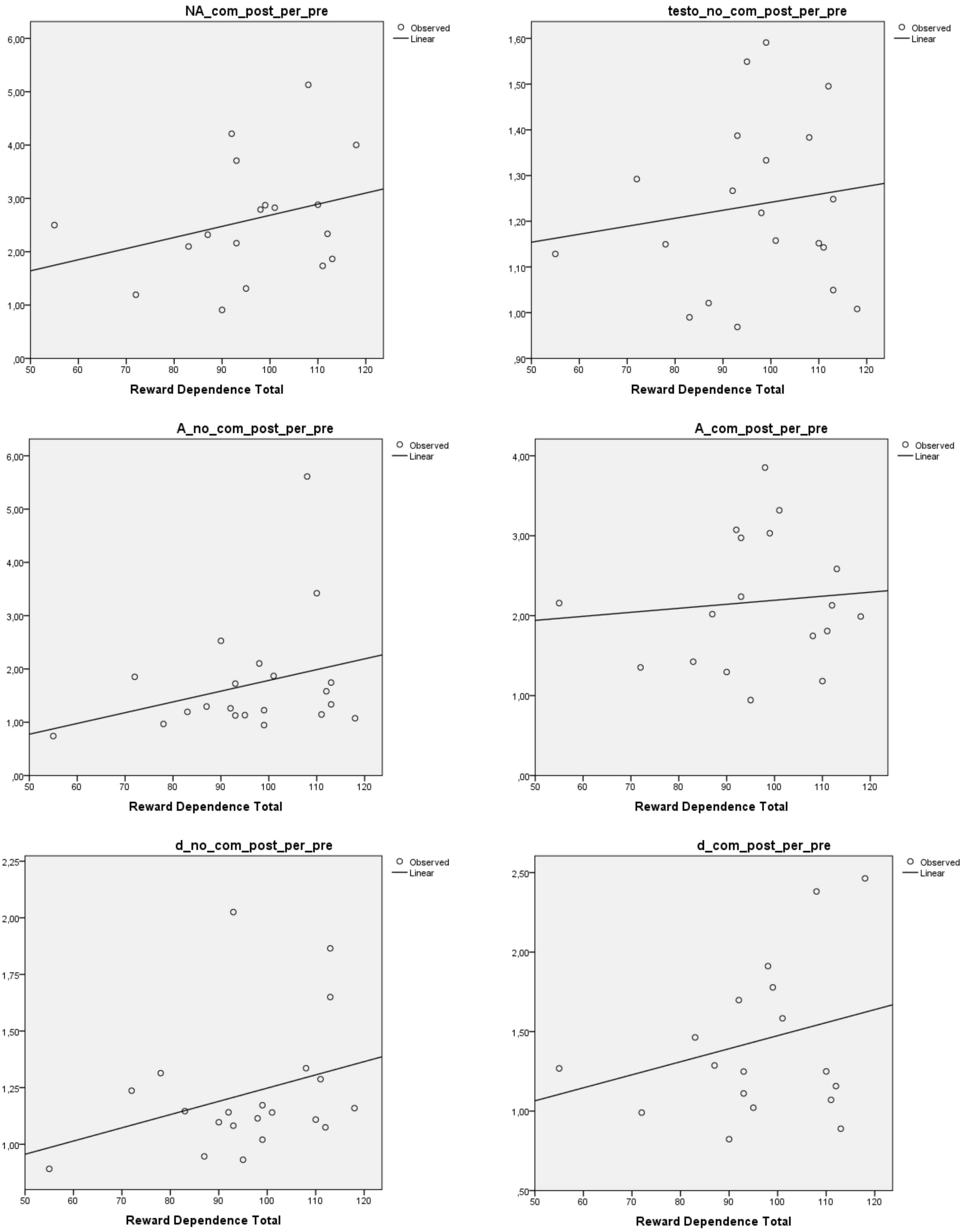

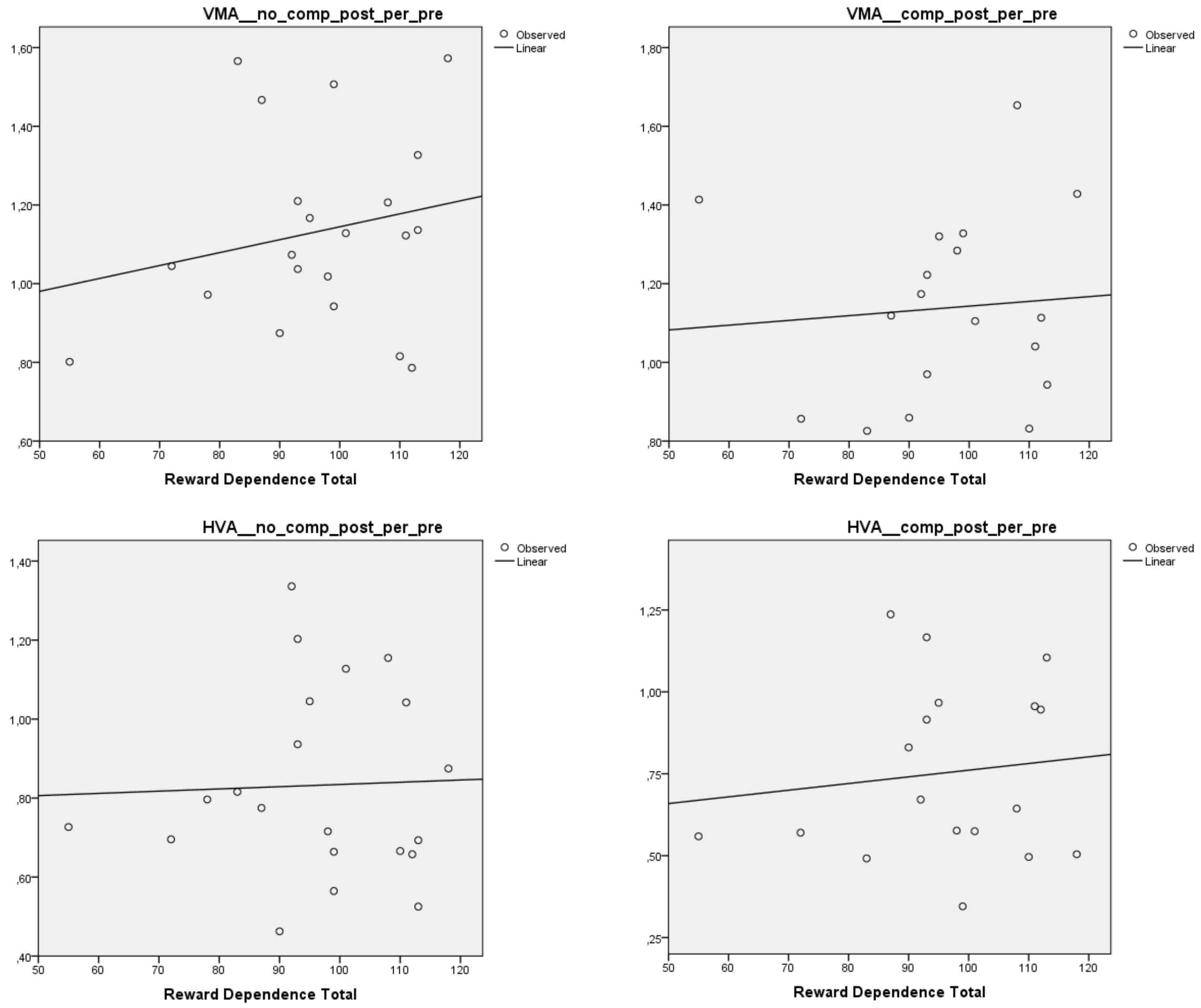

Supplement/ Figure. 2. Non-significant associations of measured biomarkers with RD scores. Non-competitive and competitive post/pre ratios were the objects of the analysis.
