# Supplementary material for: Reward Dependence-Moderated Noradrenergic and Hormonal Responses During Noncompetitive and Competitive Physical Activities
Source: Front Behav Neurosci. 2022 Apr 26;16:763220. doi: 10.3389/fnbeh.2022.763220 (PMC9087724; doi:10.3389/fnbeh.2022.763220)
Supplement: Supplementary file 3 [file Table_2.docx]

| Conditions and  CATs, hormones | n | means (SD)  pre-exercise post-exercise | t- | p | Cohen’s d |
| --- | --- | --- | --- | --- | --- |
| Non-competitive  RT |  |  |  |  |  |
| NA | 21 | 24.61 (10.7) 43.62 (19.4) | -4.73 | **0.001** | 1.03 |
| A | 21 | 7.77 (2.8) 13.82 (13.0) | -2.38 | **0.027** | 0.52 |
| D | 21 | 100.37 (25.7) 120.15 (30.1) | -4.15 | **0.001** | 0.91 |
| TE | 20 | 14.91 (6.5) 17.90 (7.5) | -5.39 | **0.001** | 1.20 |
| Non-competitive  CAT’s Metabolites |  |  |  |  |  |
| VMA | 21 | 1.40 (0.3) 1.57 (0.4) | -2.44 | **0.024** | **0.53** |
| HVA | 21 | 1.77 (0.6) 1.42 (0,4) | 3.04 | **0.006** | 0.66 |
| Competitive RT |  |  |  |  |  |
| NA | 18 | 23.05 (12.1) 55.27 (24.4) | -5.96 | **0.001** | 1.4 |
| A | 18 | 8.67 (6.1) 16.28 (9.3) | -5.95 | **0.001** | 1.4 |
| D | 18 | 108.51 (34.2) 145.94 (49.3) | -3.38 | **0.004** | 0.79 |
| TE | 18 | 14.24 (6.9) 15.41 (6.8) | -2.05 | 0.056 | 0.48 |
| Competitive  CAT’s metabolites |  |  |  |  |  |
| VMA | 18 | 1.62 (0.5) 1.81 (0.5) | -1.90 | 0.74 | 0.45 |
| HVA | 18 | 2.13 (0.8) 1.57 (0.8) | 4.14 | **0.001** | 0.97 |

**SUPPLEMENT**

**C.**

**Supplement/ Table:** Descriptive characteristics and paired two-sided t test regarding neurotransmitters, metabolites, and hormones in non-competitive and competitive RT conditions, in the pre-and post-task phase of activities. The significant main effects confirmed the findings of the paired samples t-tests. The level of NA increased from pre-task to post-task, and the increase was significantly higher during the competitive condition. The effects were large in both conditions (non-competitive Cohens’s d = 1.03, competitive Cohen’s d = 1.4). The level of testosterone increased from pre-task to post-task, however, the increase was significantly higher in the non-competitive condition. This is also reflected by the Cohen’s d effect sizes: in the non-competitive condition, the effect sizes were large (1.20), whereas in the competitive condition only small (0.48). Abbreviations: NA (Noradrenaline, in nmol/L), A (Adrenaline, in nmol/L), D (Dopamine, in nmol/L), TE (serum testosterone, in nmol/L), VMA (vanillylmandelic acid, in µmol/L), HVA (homovanillic acid, in µmol/L).
